# Computed tomography perfusion-defined ischemic core predicts functional outcome after basilar artery thrombectomy

**DOI:** 10.3389/fneur.2025.1678880

**Published:** 2025-12-08

**Authors:** Pengjun Chen, Xia Li, Yechao Huang, Junguo Hui, Jie Rao, Wenya Zhang, Lijun Shang, Xiao Chen, Ruijie Gao, Jinwei Zhou, Qiaoling Ding, Shuiwei Xia, Jiansong Ji

**Affiliations:** 1 Zhejiang Key Laboratory of Imaging and Interventional Medicine, The Fifth Affiliated Hospital of Wenzhou Medical University, Lishui Hospital of Zhejiang University, Lishui, China; 2 The Second School of Clinical Medicine, Zhejiang Chinese Medical University, Hangzhou, China; 3 Department of Neurology, The Fifth Affiliated Hospital of Wenzhou Medical University, Lishui Hospital of Zhejiang University, Lishui, China; 4 Department of Radiology, Jinyun County People’s Hospital, Lishui, China; 5 Department of Radiology, Affiliated Hangzhou Xixi Hospital, Zhejiang University School of Medicine, Hangzhou, China

**Keywords:** basilar artery occlusion, computed tomography perfusion, stroke, thrombectomy, prognosis

## Abstract

**Purpose:**

This study aimed to determine the optimal threshold for computed tomography perfusion (CTP)-defined ischemic core in patients with basilar artery occlusion (BAO) that predicts functional outcome.

**Methods:**

A retrospective analysis was conducted on BAO patients who underwent endovascular thrombectomy at our stroke center between January 2018 and March 2024. Ischemic core was estimated using following thresholds: cerebral blood flow (CBF) < 10 or 15 mL/100 g/min by Syngo.via, cerebral blood volume < 1.2 mL/100 mL by Syngo.via, and time to maximum (Tmax) > 10 s by RAPID. A favorable functional outcome was defined as a modified Rankin Scale score of 0–3 at 90-day post-onset. The Posterior Circulation Alberta Stroke Program Early computed tomography Score (pc-ASPECTS) was semi-quantified to assess ischemic changes. Statistical analysis included intraclass correlation coefficient (ICC) and receiver operating characteristic analyses.

**Results:**

A total of 85 patients were enrolled, and 39 (45.9%) had a favorable functional outcome. The ICC for pc-ASPECTS based on four core approaches between junior and senior observers ranged from 0.90 to 0.96. For the classification of favorable outcome, the volume and pc-ASPECTS core estimation approach (CBF < 10 mL/100 g/min by Syngo.via) had the best performance, with the largest area under the curve of 0.86 [(95% confidence intervals, 0.78–0.94); *p* < 0.001] and 0.87 [(95% confidence intervals, 0.80–0.94); *p* < 0.001], with a cut-off value of ≤ 2.2 (78.3%% sensitivity, 84.6% specificity), and ≥ 7 (92.3% sensitivity, 65.2% specificity).

**Conclusion:**

In BAO patients following successful recanalization, the volume and pc-ASPECTS core estimation approach (CBF < 10 mL/100 g/min by Syngo.via) demonstrated the strongest predictive value for favorable functional outcomes.

## 1 Introduction

Delineation of the location, profile and size of the ischemic core defined by Computed Tomography perfusion (CTP) has become standard practice in managing anterior circulation acute ischemic stroke caused by large artery occlusion (LVO) (1–4), and this assessment is pivotal for determining the functional outcomes (5–8). Typically, in patients with anterior circulation LVO, a relative cerebral blood flow (rCBF) of less than 30% accurately reflects the final infarct profile after intravenous thrombolysis, while an rCBF volume of less than 20% is the closest predictor of final infarct volume post-endovascular treatment (3, 9–14). However, the conventional thresholds used to identify the ischemic core may not apply to patients with acute basilar artery occlusion (BAO), due to the unique bilateral hypoperfusion or infarction pattern resulting from specific blood supply of the basilar artery (15–17). Thus, a novel approach with an optimal threshold is needed for identifying the ischemic core in BAO patients.

Previous studies indicate that normal brain mixed cortical flows averaged approximately 51 mL/100 g/min (18, 19). Based on this, we hypothesize that absolute cerebral blood flow (CBF) values of less than 10 mL/100 g/min (corresponding to rCBF < 20%) and less than 15 mL/100 g/min (corresponding to rCBF < 30%) are promising threshold candidates for identifying the ischemic core in BAO patients.

Additionally, the Siemens Syngo.via software recommends an ischemic core estimation approach using a cerebral blood volume (CBV)-based threshold of less than 1.2 mL/100 mL for patients with anterior circulation LVO (20). However, this threshold has yet to be validated in BAO patients. Furthermore, Yuen found that the volume of time to maximum (Tmax) greater than 10 s, generated by RAPID software, correlated well with final infarct volume in BAO patients after successful reperfusion (21).

Therefore, our aim is to determine the optimal threshold for predicting favorable functional outcome among four ischemic core estimation approaches, including CBF < 10 mL/100 g/min by Syngo.via software, CBF < 15 mL/100 g/min by Syngo.via software, CBV < 1.2 mL/100 mL by Syngo.via software, and Tmax > 10 s by RAPID software.

## 2 Methods

### 2.1 Study population

Ethics approval was obtained from the institutional review board of Lishui Central Hospital (approval number, 2022357), and the study complied with the guidelines of the Declaration of Helsinki. Requirement for written informed consent was waived by our review boards due to the retrospective nature of the study.

We performed a single-center retrospective study of BAO patients who underwent CTP scanning prior to EVT. Patients were enrolled from January 2018 to March 2024.

The inclusion criteria for eligible patients in this study were as follows: (1) age ≥ 18 years with a National Institutes of Health Stroke Scale (NIHSS) score of 6 or higher; (2) a standard CT protocol that included noncontrast computed tomography (NCCT) and CTP examination prior to EVT upon admission, revealing significant perfusion deficits; (3) confirmation of BAO on reconstructed computed tomography angiography (CTA); and (4) execution of EVT.

The exclusion criteria were as follows: (1) premorbid modified Rankin Scale (mRS) of 3 or higher; (2) stroke recurrence within a 90-day period; (3) time from symptom onset to EVT puncture more than 24 h; (4) severe cardiopulmonary insufficiency; and (5) non-interpretable CTP imaging due to the poor quality.

A ‘significant perfusion deficit’ was defined as a visually detectable decrease in CBF or CBV maps, or an increase in Tmax maps, observed on at least two adjacent slices.

### 2.2 Imaging protocol

The imaging acquisition protocol was consistent with that described in previous studies (16). For all patients admitted, a standardized CT protocol was executed consisting of NCCT and CTP, extending from the base of the skull to the parietal lobe. NCCT imaging data was acquired at 100 kVp and 310 mAs, with a slice thickness of 1 mm and reconstructed at 5 mm. Following an initial 4 s delay, CTP data were collected every 1.5–3 s for a duration 51 s. The scan was processed at 80 kVp and 100 mAs, after an intravenous injection of 50 mL contrast at a rate of 8 mL/s.

### 2.3 Data processing

Color-coded perfusion maps, including CBF, CBV, and Tmax, was obtained with a slice thickness of 5 mm every 3 mm.

Four methods for identifying presumed ischemic cores, generated by either Syngo.via or RAPID software, were investigated: Approach 1 represents CBF < 10 mL/100 g/min (equivalent to rCBF < 20%) maps generated by Syngo.via software. Approach 2 represents CBF < 15 mL/100 g/min (equivalent to rCBF < 30%) maps generated by Syngo.via software. Approach 3 represents CBV < 1.2 mL/100 mL maps generated by Syngo.via software. Approach 4 represents Tmax > 10 s maps generated by RAPID software. These thresholds were selected due to their common usage or recommendation in previous studies and daily clinical practice.

For CTA analysis, slices were reconstructed with a thickness of 0.625 mm, spaced every 1 mm, from the peak phase of the time-attenuation curve. CT attenuation was measured by placing a circular region of interest in the proximal middle cerebral artery on axial images.

### 2.4 Baseline imaging assessment

The extent of arterial occlusions, collateral circulation, and anatomic variants were evaluated using three CTA-based markers: the posterior circulation collateral score (pc-CS) (22), the posterior circulation computed tomography angiography (pc-CTA) score (23), and the basilar artery on computed tomography angiography (BATMAN) prognostic score (24). Additionally, the location of the occlusions, the presence of hyperdense basilar artery sign, and vertebrobasilar artery atherosclerosis were also documented.

To assess early ischemic changes, NCCT images, CTA source images (CTA-SI), color-coded CTP maps, presumed ischemic core maps (approach 1 to 4), and follow-up imaging were evaluated using the Posterior circulation Alberta Stroke Program Early computed tomography Score (pc-ASPECTS) (25). A region with at least one diameter of at least 6 mm was considered as positive for ischemia. The pc-ASPECTS is a 10-point score that decreases if ischemic changes occur in specific brain regions: 1 point is subtracted for unilateral involvement of the cerebellum, thalamus, or posterior cerebral artery territory, and 2 points are deducted for involvement of the pons and midbrain.

The qualitative and semi-quantitative assessment of all imaging data was performed by two independent readers: a junior radiologist with 6-year experience and a senior neuro-radiologist with 13-year experience. Both readers were blinded to the clinical data and outcomes. In cases of disagreement, a separate session was held to reach a final decision, with the participation of a third senior neuro-radiologist, who has 20-year experience.

### 2.5 Clinical data

Trained investigators, who were blinded to all imaging data and follow-up outcomes, collected the following clinical information: age, sex, coma status upon admission, baseline NIHSS score, onset-to-scan time, scan-to-recanalization time, mRS scores before and 90 days after symptom onset, volume of core Approach 1 to 4, volume of Tmax > 8 s, 6 s, and 4 s generated by RAPID software, risk factors, and treatment data.

Prior to EVT, eligible patients were administered intravenous thrombolysis treatment. EVT was the primary treatment option, with balloon angioplasty and/or stenting being selected as appropriate to achieve successful recanalization, defined as a modified Thrombolysis in Cerebral Infarction (mTICI) score of 2b-3. A favorable functional outcome was considered as an mRS score of 0–3, assessed 90 days after EVT.

### 2.6 Statistical analysis

Statistical analysis was done using MedCalc Statistical Software version 20.022 and SPSS Statistics 27. Continuous variables were described by mean ± standard deviation, and compared by Mann–Whitney U test. While, categorical variables were expressed as counts and percentages, and compared using chi-square test.

Imaging parameters with a *p*-value < 0.10 in univariate analysis were individually subjected to multivariate binary logistic regressions separately. Baseline covariates included age, NIHSS scores, and the presence of coma upon admission. Receiver Operating Characteristic (ROC) analyses were performed to differentiate between good and poor outcomes, with metrics including the area under the curve (AUC), sensitivity, and specificity. Optimal cutoff values were determined by using the Youden method.

The intraclass correlation coefficient (ICC) was utilized to evaluate the inter-rater reliability between junior and senior readers in assessing the pc-ASPECTS scale.

Statistical significance was defined as *p* < 0.05.

## 3 Results

### 3.1 Study population

**Figure 1:**
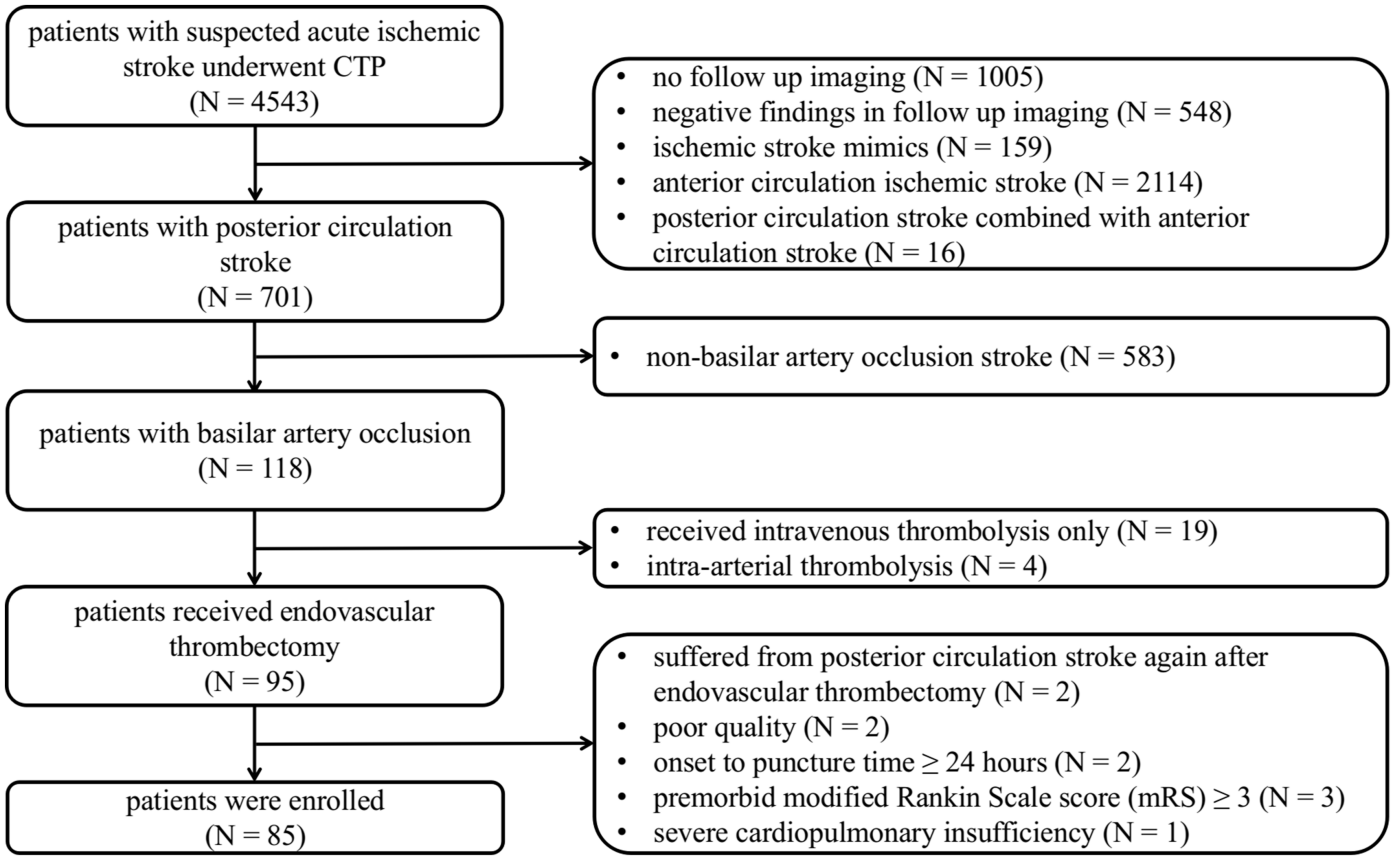
Flow chart of the study population. CTP, CT perfusion.

The flowchart illustrating the enrollment process is depicted in Figure 1. From January 2018 to March 2024, 4,543 symptomatic patients with suspected acute ischemic stroke were referred for brain NCCT and CTP. Of which, 701 patients suffered with posterior circulation stroke diagnosed by follow-up imaging, and 118 patients were confirmed with BAO. This study excluded 19 patients who underwent intravenous thrombolysis only, 4 patients who underwent intra-arterial thrombolysis, 3 patients with premorbid mRS 3 or higher, 1 patient with severe cardiopulmonary insufficiency, 2 patients with onset to puncture time more than 24 h, and 2 patients with poor image quality. A total of 85 patients (mean age 67.2 ± 11.3 years, 72.9% men) with BAO who underwent EVT were included in our study. Of all patients, 54 (63.5%) underwent MRI-DWI as follow-up imaging and 31 (36.5%) underwent NCCT only due to contraindications.

Among all patients, 43 (50.6%) exhibited prodromal symptoms, and 46 (54.1%) were in a coma upon admission. The mean baseline NIHSS score was 22.8 ± 8.9, ranged from 7 to 40. Prior to EVT, either at a primary care center or our stroke center, intravenous thrombolysis was administered to 19 patients (22.4%). Regarding treatment modalities, 56 (65.9%) patients underwent EVT alone, while 29 (34.1%) underwent EVT combined with balloon angioplasty and/or stenting. Additionally, 22 (25.9%) patients underwent tandem EVT. Successful recanalization was achieved in all patients, with 75 (88.2%) patients achieved an mTICI score of 3, and 10 (11.8%) patients achieved an mTICI score of 2b. The mean onset-to-scan time was 401.9 ± 206.6 min, with a range of 63 to 1,138 min. The mean scan-to-puncture was 97.4 ± 35.7 min, varying between 34 and 197 min. Additionally, the mean puncture-to-recanalization time was 95.9 ± 34.0 min, ranging from 27 to 184 min.

In terms of functional outcomes, 39 (45.9%) patients had a favorable functional outcome, and 22 (25.9%) died. Patients with a favorable outcome had a lower incidence of coma upon admission (*p* < 0.001), a lower baseline NIHSS score (*p* < 0.001), a shorter onset-to-scan time (*p* = 0.03), and a limited volume of volume of core Approach 1 to 4 (*p* < 0.001), Tmax > 4 s (*p* = 0.04), and 8 s (*p* < 0.01) as assessed by RAPID software. They also lacked the hyperdense basilar artery sign (*p* < 0.01). Higher pc-ASPECTS based on NCCT, color-coded CTP maps, and all approaches ischemic core approaches were associated with a favorable outcome, except for pc-ASPECTS based on color-coded Tmax maps. However, no significant difference were observed between the two groups for any CTA-based markers (*p* > 0.05). The baseline characteristics of the favorable and poor functional outcome groups are detailed in Tables 1, 2.

**Table 1:** Patients data.

Variables	Overall (*n* = 85)	mRS 0–3 (*n* = 39)	mRS 4–6 (*n* = 46)	*p* value
Age, years	67.2 ± 11.3	67.0 ± 11.7	67.3 ± 11.1	1.00
Male sex, *n* (%)	62 (72.9%)	25 (64.1%)	37 (80.4%)	0.09
Hypertension, *n* (%)	64 (75.3%)	30 (76.9%)	34 (73.9%)	0.75
Diabetes mellitus, *n* (%)	32 (37.6%)	13 (33.3%)	19 (41.3%)	0.45
Hyperlipidemia, *n* (%)	13 (15.3%)	4 (10.3%)	9 (19.6%)	0.24
Atrial fibrillation, *n* (%)	10 (11.8%)	5 (12.8%)	5 (10.9%)	0.78
Coronary artery disease, *n* (%)	8 (9.4%)	4 (10.3%)	4 (8.7%)	0.81
Smoking, *n* (%)	26 (30.6%)	13 (33.3%)	13 (28.3%)	0.61
Alcohol abuse, *n* (%)	12 (14.1%)	5 (12.8%)	7 (15.2%)	0.75
History of infarcts, *n* (%)	8 (9.4%)	6 (15.4%)	2 (4.3%)	0.08
Prodromal symptoms, *n* (%)	43 (50.6%)	22 (56.4%)	21 (45.7%)	0.32
Coma on admission, *n* (%)	46 (54.1%)	12 (30.8%)	34 (73.9%)	< 0.001*
NIHSS score	22.8 ± 8.9	17.8 ± 8.0	27.0 ± 7.4	< 0.001*
onset-to-scan time, min	401.9 ± 206.6	367.2 ± 213.1	431.4 ± 198.4	0.03*
scan-to-puncture time, min	97.4 ± 35.7	101.1 ± 37.2	94.3 ± 34.5	0.34
puncture-to-recanalization time, min	95.9 ± 34.0	90.5 ± 35.3	100.5 ± 32.6	0.13
Intravenous thrombolysis, *n* (%)	19 (22.4%)	10 (25.6%)	9 (19.6%)	0.50
EVT alone, *n* (%)	29 (34.1%)	12 (30.8%)	17 (37.0%)	0.55
Tandem EVT, *n* (%)	22 (25.9%)	10 (25.6%)	12 (26.1%)	0.96
mTICI 3, *n* (%)	75 (88.2%)	34 (87.2%)	46 (89.1%)	0.78
mTICI 2b, *n* (%)	10 (11.8%)	5 (12.8%)	5 (10.9%)	0.78
Premobid mRS	0.2 ± 0.4	0.2 ± 0.5	0.2 ± 0.4	0.54
90-day mRS	3.6 ± 2.0	1.7 ± 1.2	5.3 ± 0.8	–

Values presented are number (percentage) for categorical variables, and mean for ordinal and continuous variables. mRS, modified Rankin Scale; NIHSS, National Institutes of Health Stroke Scale; EVT, endovascular thrombectomy; mTICI, modified Thrombolysis In Cerebral Infarction. *p values indicate *p* < 0.05.

**Table 2:** Imaging data.

Imaging data	Overall (*n* = 85)	mRS 0–3 (*n* = 39)	mRS 4–6 (*n* = 46)	*p* value
Hyperdense basilar artery sign, *n* (%)	69 (81.2%)	27 (69.2%)	42 (91.3%)	< 0.01*
Vertebrobasilar artery atherosclerosis, *n* (%)	66 (77.6%)	32 (82.1%)	34 (73.9%)	0.37
Vetebral arteries, *n* (%)	28 (32.9%)	11 (28.2%)	17 (37.0%)	0.39
Proximal basilar artery, *n* (%)	51 (60.0%)	21 (53.8%)	30 (65.2%)	0.29
Middle basilar artery, *n* (%)	71 (83.5%)	28 (71.8%)	43 (93.5%)	< 0.01*
Distal basilar artery, *n* (%)	51 (60.0%)	25 (64.1%)	26 (56.5%)	0.48
BATMAN	5.6 ± 1.6	5.9 ± 1.5	5.3 ± 1.6	0.15
pc-CTA	3.0 ± 1.2	3.2 ± 1.2	2.9 ± 1.2	0.27
pc-CS	5.8 ± 1.6	6.2 ± 1.7	5.5 ± 1.4	0.08
pc-ASPECTS NCCT	8.2 ± 1.5	8.8 ± 1.2	7.7 ± 1.6	< 0.01*
pc-ASPECTS CBF	3.7 ± 2.0	4.6 ± 1.8	2.9 ± 1.8	< 0.001*
pc-ASPECTS CBV	7.0 ± 1.7	8.2 ± 1.3	6.0 ± 1.4	< 0.001*
pc-ASPECTS Tmax	2.0 ± 2.3	2.5 ± 2.5	1.50 ± 2.1	0.07
pc-ASPECTS Approach 1	6.7 ± 2.0	8.1 ± 1.3	5.6 ± 1.8	< 0.001*
pc-ASPECTS Approach 2	4.9 ± 2.3	5.8 ± 2.2	4.2 ± 2.1	< 0.01*
pc-ASPECTS Approach 3	7.0 ± 2.0	7.9 ± 1.6	6.1 ± 2.0	< 0.001*
pc-ASPECTS Approach 4	7.6 ± 2.4	8.6 ± 2.0	6.7 ± 2.3	< 0.01*
Volume of core Approach 1, mL	4.8 ± 6.8	1.5 ± 1.2	7.5 ± 8.2	< 0.001*
Volume of core Approach 2, mL	12.3 ± 12.0	5.7 ± 4.1	17.9 ± 13.6	< 0.001*
Volume of core Approach 3, mL	6.6 ± 9.0	2.5 ± 2.8	10.0 ± 10.9	< 0.001*
Volume of core Approach 4, mL	14.0 ± 19.5	7.9 ± 14.8	19.1 ± 21.5	< 0.001*
Tmax > 8 s volume by RAPID, mL	30.9 ± 30.6	24.7 ± 32.0	36.2 ± 28.7	< 0.01*
Tmax > 6 s volume by RAPID, mL	71.7 ± 49.3	64.6 ± 57.1	77.7 ± 41.2	0.08
Tmax > 4 s volume by RAPID, mL	125.4 ± 70.7	113.0 ± 73.8	136.0 ± 66.9	0.04*
Follow-up pc-ASPECTS	5.8 ± 2.0	7.0 ± 1.6	4.8 ± 1.8	< 0.001*

mRS, modified Rankin Scale; BATMAN, basilar artery on CT angiography; pc-CTA, posterior circulation CT angiography; pc-CS, posterior circulation collateral score; pc-ASPECTS, Posterior Circulation Alberta Stroke Program Early CT Score; NCCT, noncontrast CT; CBF, cerebral blood flow; CBV, cerebral blood volume; Tmax, time to maximum. Approach 1 represents CBF < 10 mL/100 g/min (rCBF < 20%) maps generated by Syngo.via software. Approach 2 represents CBF < 15 mL/100 g/min (rCBF < 30%) maps generated by Syngo.via software. Approach 3 represents CBV < 1.2 mL/100 mL maps generated by Syngo.via software. Approach 4 represents Tmax > 10 s maps generated by RAPID software. *p values indicate *p* < 0.05.

For the pc-ASPECTS data based on the presumed ischemic core approaches 1 to 4, the ICCs between a senior neuro-radiologist and a junior radiologist ranged from 0.90 to 0.96, indicating an excellent agreement in score assignment. Additionally, for the pc-ASPECTS data based on NCCT, CBF, and CBV, the ICCs were 0.74, 0.75, and 0.65, respectively (see Table 3).

**Table 3:** The inter-observer reliability in score assignment between professional senior neuro-radiologist and primary junior radiologist.

Independent variables	pc-ASPECTS by junior observer	pc-ASPECTS by senior observer	ICC (95% CI)
pc-ASPECTS NCCT	8.5 ± 1.6	8.2 ± 1.5	0.74 (0.61–0.83)
pc-ASPECTS CBF	3.3 ± 2.2	3.7 ± 2.0	0.75 (0.63–0.83)
pc-ASPECTS CBV	6.2 ± 2.1	7.0 ± 1.7	0.65 (0.39–0.79)
pc-ASPECTS Tmax	2.1 ± 2.4	2.0 ± 2.3	0.91 (0.87–0.94)
pc-ASPECTS Approach 1	7.0 ± 1.9	6.7 ± 2.0	0.90 (0.84–0.94)
pc-ASPECTS Approach 2	5.0 ± 2.1	4.9 ± 2.3	0.90 (0.85–0.93)
pc-ASPECTS Approach 3	6.8 ± 2.0	7.0 ± 2.0	0.90 (0.85–0.93)
pc-ASPECTS Approach 4	7.7 ± 2.3	7.6 ± 2.4	0.96 (0.94–0.97)

pc-ASPECTS, Posterior Circulation Alberta Stroke Program Early CT Score; ICC, intraclass correlation coefficient; CI, confidence interval; NCCT, noncontrast CT; CBF, cerebral blood flow; CBV, cerebral blood volume; Tmax, time to maximum. Approach 1 represents CBF < 10 mL/100 g/min (rCBF < 20%) maps generated by Syngo.via software. Approach 2 represents CBF < 15 mL/100 g/min (rCBF < 30%) maps generated by Syngo.via software. Approach 3 represents CBV < 1.2 mL/100 mL maps generated by Syngo.via software. Approach 4 represents Tmax > 10 s maps generated by RAPID software.

### 3.2 Imaging parameters for favorable outcome prediction

The results of the multivariable logistic analysis are shown in Table 4. Following the collinearity diagnosis, adjustments were made for the potential confounders (*p* < 0.10) such as male sex, history of infarcts, coma on admission, NIHSS score, onset-to-scan time, hyperdense basilar artery sign, middle basilar artery, and pc-CS. Despite these adjustments, pc-ASPECTS NCCT, pc-ASPECTS CBF, pc-ASPECTS CBV, pc-ASPECTS Approach 1, pc-ASPECTS Approach 4, and volume of core Approach 1 to 3 continued to serve as independent predictors of favorable outcomes at 90-days.

**Table 4:** Prediction of image data for Favorable functional outcome.

Independent variables	Favorable functional outcome	
	OR (95% CI)	*p* value
pc-ASPECTS NCCT	0.57 (0.34, 0.95)	0.03*
pc-ASPECTS CBF	0.54 (0.35, 0.83)	< 0.01*
pc-ASPECTS CBV	0.34 (0.20, 0.61)	< 0.001*
pc-ASPECTS Approach 1	0.20 (0.08, 0.51)	0.001*
pc-ASPECTS Approach 2	0.75 (0.55, 1.03)	0.07
pc-ASPECTS Approach 3	0.71 (0.49, 0.91)	0.06
pc-ASPECTS Approach 4	0.67 (0.49, 0.92)	0.01*
Volume of core Approach 1	2.53 (1.33, 4.83)	< 0.01*
Volume of core Approach 2	1.26 (1.08, 1.48)	< 0.01*
Volume of core Approach 3	1.24 (1.04, 1.48)	0.02*
Volume of core Approach 4	1.03 (1.00, 1.07)	0.15
Tmax > 8 s volume by RAPID	1.00 (0.98, 1.03)	0.69
Tmax > 4 s volume by RAPID	1.00 (1.00, 1.01)	0.52

Favorable functional outcome, 90-d modified Rankin Scale (mRS) score 0–3. OR, odds ratio; CI, confidence interval; pc-ASPECTS, Posterior Circulation Alberta Stroke Program Early CT Score; NCCT, noncontrast CT; CBF, cerebral blood flow; CBV, cerebral blood volume; Tmax, time to maximum. Approach 1 represents CBF < 10 mL/100 g/min (rCBF < 20%) maps generated by Syngo.via software. Approach 2 represents CBF < 15 mL/100 g/min (rCBF < 30%) maps generated by Syngo.via software. Approach 3 represents CBV < 1.2 mL/100 mL maps generated by Syngo.via software. Approach 4 represents Tmax > 10 s maps generated by RAPID software. *p values indicate *p* < 0.05.

**Figure 2:**
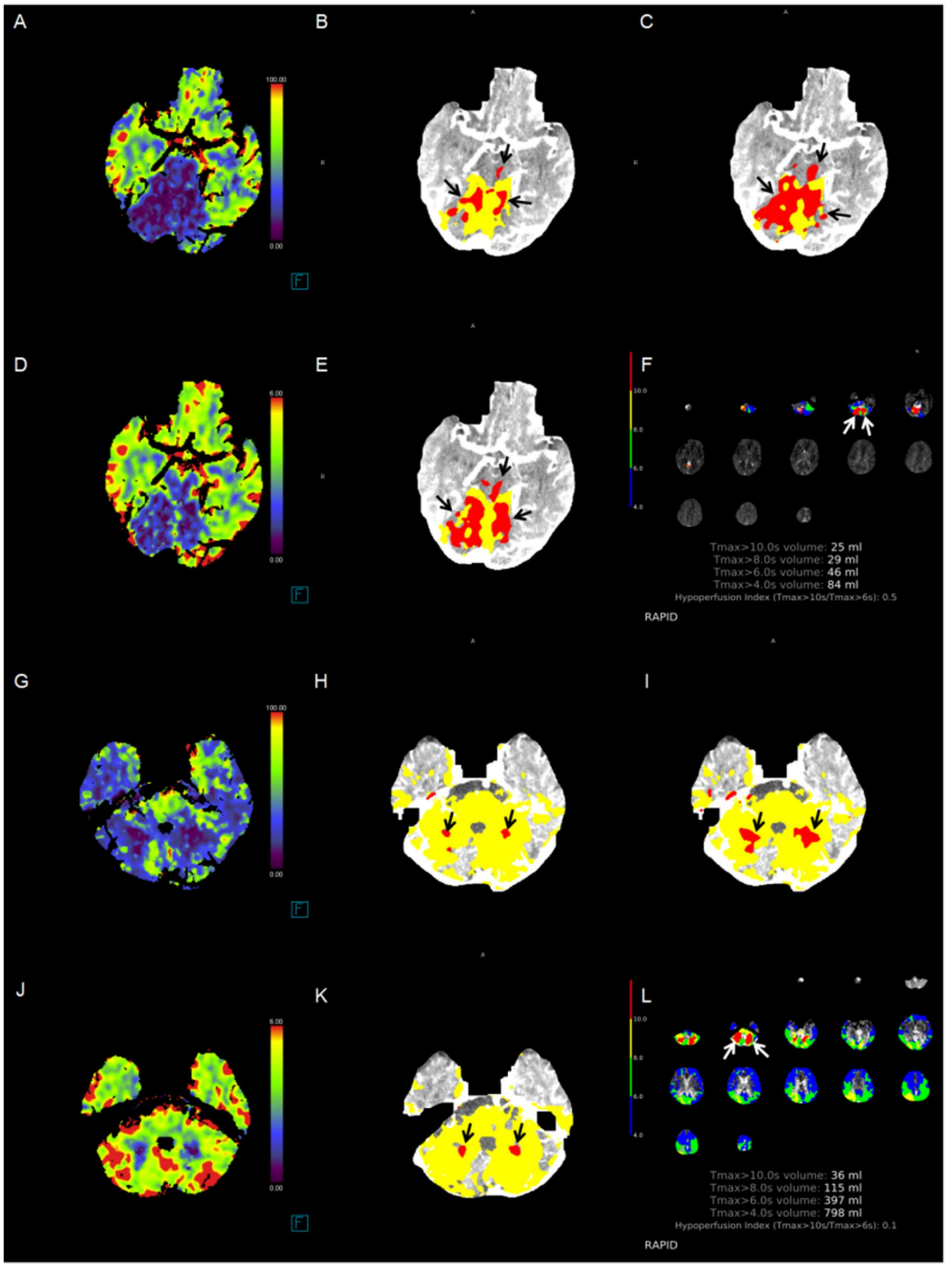
Case examples. The correlation between the cerebral blood flow absolute value-based ischemic core extent and functional outcome is more significant. Red area indicates the ischemic core (black arrow). Patient 1: A patient had an initial time to maximum (Tmax) > 10 s for a volume of 25 mL in the bilateral cerebellar hemisphere, as identified by RAPID software, due to basilar artery occlusion. **(A)** ischemic lesions in the pons and bilateral cerebellum, pc-ASPECTS CBF was 6; **(B)** pc-ASPECTS Approach 1 was 6; **(C)** pc-ASPECTS Approach 2 was 6; **(D)** pc-ASPECTS CBV was 6; **(E)** pc-ASPECTS Approach 3 was 6; **(F)** ischemic lesions in bilateral cerebellum, pc-ASPECTS Approach 4 was 8. The 90-day modified Rankin Scale score was 5. Patient 2: A patient had an initial time to maximum (Tmax) > 10 s for a volume of 36 mL in the bilateral cerebellar hemisphere, as identified by RAPID software, due to basilar artery occlusion. **(G)** ischemic lesions in bilateral cerebellum, pc-ASPECTS CBF was 8; **(H)** pc-ASPECTS Approach 1 was 8; **(I)** pc-ASPECTS Approach 2 was 8; **(J)** pc-ASPECTS CBV was 8; **(K)** pc-ASPECTS Approach 3 was 6; **(L)** pc-ASPECTS Approach 4 was 8. All pc-ASPECTS were 8. The 90-day modified Rankin Scale score was 2. pc-ASPECTS, Posterior Circulation Alberta Stroke Program Early CT Score. Approach 1 represents CBF < 10 mL/100 g/min (rCBF < 20%) maps generated by Syngo.via software. Approach 2 represents CBF < 15 mL/100 g/min (rCBF < 30%) maps generated by Syngo.via software. Approach 3 represents CBV < 1.2 mL/100 mL maps generated by Syngo.via software. Approach 4 represents Tmax > 10 s maps generated by RAPID software.

**Figure 3:**
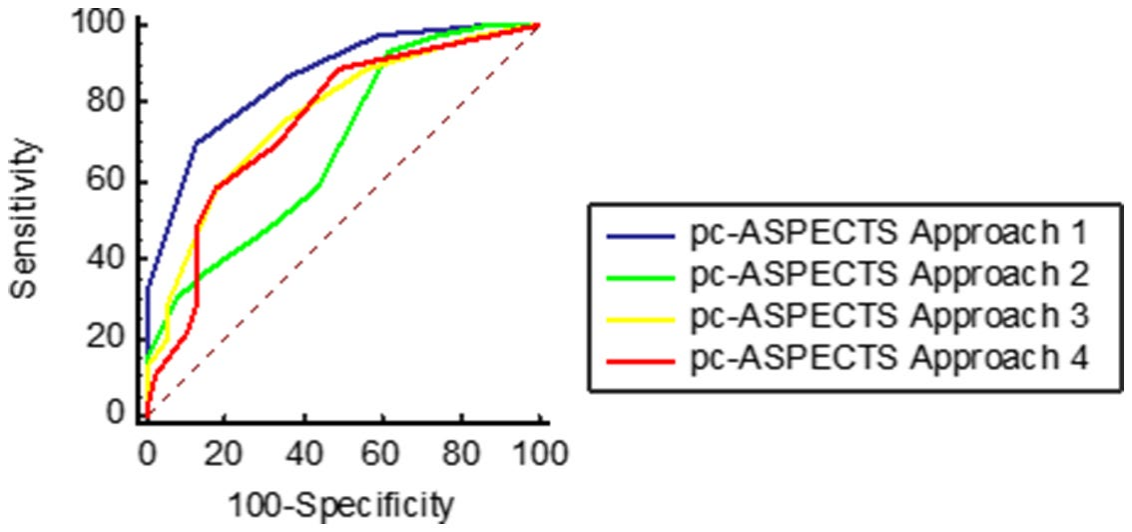
Receiver operating characteristics analysis of pc-ASPECTS Approach 1 to 4 to identify patients with a favorable functional outcome. Approach 1 represents CBF < 10 mL/100 g/min (rCBF < 20%) maps generated by Syngo.via software. Approach 2 represents CBF < 15 mL/100 g/min (rCBF < 30%) maps generated by Syngo.via software. Approach 3 represents CBV < 1.2 mL/100 mL maps generated by Syngo.via software. Approach 4 represents Tmax > 10 s maps generated by RAPID software.

**Figure 4:**
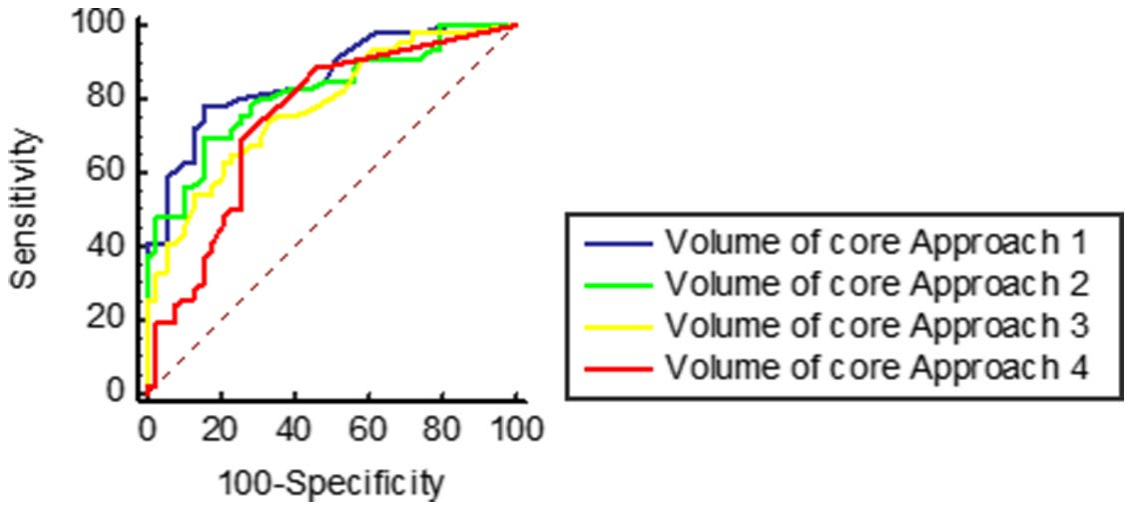
Receiver operating characteristics analysis of volume of core Approach 1 to 4 to identify patients with a favorable functional outcome. Approach 1 represents CBF < 10 mL/100 g/min (rCBF < 20%) maps generated by Syngo.via software. Approach 2 represents CBF < 15 mL/100 g/min (rCBF < 30%) maps generated by Syngo.via software. Approach 3 represents CBV < 1.2 mL/100 mL maps generated by Syngo.via software. Approach 4 represents Tmax > 10 s maps generated by RAPID software.

In ROC analyses, pc-ASPECTS approach 1, volume of core Approach 1, and pc-ASPECTS CBV demonstrated excellent performance, with the largest AUC of 0.87 [(95% confidence intervals CI, 0.79–0.94); *p* < 0.001], 0.86 [(95% CI, 0.78–0.94); *p* < 0.001], and 0.86 [(95% CI, 0.78–0.94); *p* < 0.001] respectively. The optimal cut-off values were ≥ 7 for pc-ASPECTS approach 1 (with a sensitivity of 87.2% and specificity of 69.6%), ≤ 2.2 (78.3%% sensitivity, 84.6% specificity), and ≥ 8 for pc-ASPECTS CBV (with a sensitivity of 76.9% and specificity of 87.0%). These values surpassed those obtained from pc-ASPECTS based on other ischemic core approach maps (see Table 5 for details). Figure 2 illustrates examples of the prognostic differentiation capability of ischemic core across 4 promising thresholds. Figures 3, 4 showed the pc-ASPECTS and volume of core Approach 1 to 4 to identify patients with a favorable functional outcome.

**Table 5:** Receiver operating characteristics analysis of image parameters for favorable functional outcome prediction.

Variables	Favorable functional outcome					
	AUC (95% CI)	*p* value	Youden index	Cutoff value	Sensitivity	Specificity
NIHSS score	0.75 (0.71–0.89)	< 0.001*	0.47	≤ 26	65.2%	82.1%
pc-ASPECTS CBF	0.73 (0.62–0.84)	< 0.001*	0.33	≥ 3	84.6%	47.8%
pc-ASPECTS CBV	0.86 (0.78–0.94)	< 0.001*	0.64	≥ 8	76.9%	87.0%
pc-ASPECTS approach 1	0.87 (0.79–0.94)	< 0.001*	0.57	≥ 7	87.2%	69.6%
pc-ASPECTS approach 2	0.69 (0.58–0.80)	< 0.01*	0.28	≥ 7	38.5%	89.1%
pc-ASPECTS approach 3	0.77 (0.67–0.87)	< 0.001*	0.23	≥ 8	25.6%	97.8%
pc-ASPECTS approach 4	0.75 (0.65–0.86)	< 0.001*	0.41	≥ 8	82.1%	58.7%
Volume of core approach 1	0.86 (0.78–0.94)	< 0.001*	0.63	≤ 2.2	78.3%	84.6%
Volume of core approach 2	0.82 (0.73–0.91)	< 0.001*	0.52	≤ 8.3	67.4%	84.6%
Volume of core approach 3	0.78 (0.68–0.88)	< 0.001*	0.43	≤ 3.9	63.0%	79.5%
Volume of core approach 4	0.78 (0.64–0.86)	< 0.001*	0.44	≤ 5.5	69.6%	74.4%
follow-up pc-ASPECTS	0.82 (0.73–0.91)	< 0.001*	0.50	≥ 7	59.0%	91.3%

Favorable functional outcome, 90-d modified Rankin Scale (mRS) score 0–3. AUC, areas under the curve; CI, confidence interval; NIHSS, National Institutes of Health Stroke Scale; pc-ASPECTS, Posterior Circulation Alberta Stroke Program Early CT Score; CBF, cerebral blood flow; CBV, cerebral blood volume. Approach 1 represents CBF < 10 mL/100 g/min (rCBF < 20%) maps generated by Syngo.via software. Approach 2 represents CBF < 15 mL/100 g/min (rCBF < 30%) maps generated by Syngo.via software. Approach 3 represents CBV < 1.2 mL/100 mL maps generated by Syngo.via software. Approach 4 represents Tmax > 10 s maps generated by RAPID software. *p values indicate *p* < 0.05.

## 4 Discussion

This study shows that the pc-ASPECTS and volume based on ischemic core estimation approach, using a CBF threshold of < 10 mL/100 g/min (rCBF < 20%) generated by Syngo.via software, serves as a more powerful predictor of 90-day functional outcome in BAO patients following successful recanalization. There was excellent agreement in score assignment between a professional senior neuro-radiologist and a primary junior radiologist. Notably, despite a higher mean baseline NIHSS score (22 vs. a range of 17 to 21), our cohort’s favorable outcome (90-day mRS 0–3) rate was comparable to those observed in recent randomized controlled trials (45.9% vs. 42–46%). Additionally, our cohort exhibited a lower mortality rate (25.9% vs. 31–38%) (26–30). A key distinction in our research was the frequent use of balloon angioplasty and/or stenting to achieve successful recanalization. Consequently, our findings underscore the benefits and importance of successful recanalization, challenging the negative results in some trials.

Baseline predictive image data holds significant potential for guiding clinical therapy decisions. While brain MRI-diffusion weighted imaging (MRI-DWI) is regarded as the ‘golden’ standard for detecting early ischemic changes in posterior circulation strokes, CT remains widely accessible, and the Stroke Treatment Academic Industry Roundtable recommends CTP as the initial imaging strategy (31). CTP-defined ischemic core offers a more accessible and rapid alternative to MRI-DWI in patients with anterior circulation LVO, and the baseline ischemic core has proven to be a powerful predictor of functional outcome (3, 7, 9, 11, 12). However, its efficacy in strokes caused by BAO has been less studied. Various research groups have attempted to investigate the predictive value of baseline perfusion imaging parameters. Pallesen found that pc-ASPECTS based on color-coded CTP maps have predictive value in patients with BAO through visual inspection, with extensive ischemic changes on CBV maps (pc-ASPECTS < 8) associated with high case fatality (25). Alemseged extended this work and found that an initial pc-ASPECTS CBV ≤ 8 was associated with poor outcome (32). Partially aligning with their findings, our cohort exhibited significant differences in pc-ASPECTS based on NCCT, CBF, and CBV between the favorable and poor prognostic groups, suggesting that NCCT, CBF, and CBV better delineate severe ischemia. In our results, among the visual evaluations of color-coded maps, the pc-ASPECTS based on CBV maps outperformed that based on CBF maps, whereas the core approach showed the opposite trend. This is because CBF demonstrates higher sensitivity (84.6%) to severe ischemia but lower specificity (47.8%). After defining with a threshold, the CBF core approach improved its specificity to 69.6% while maintaining relatively high sensitivity. Conversely, the pc-ASPECTS based on CBV exhibited slightly lower sensitivity (76.9%) but high specificity (87.0%). However, when using the Siemens-recommended threshold of CBV < 1.2 mL/100 mL for core estimation, the sensitivity further declined (to 25.6%). This suggests that a CBV < 1.2 mL/100 mL threshold may not be suitable for the BAO (basilar artery occlusion) population.

The key disadvantage of evaluation based on NCCT, CBF, and CBV maps is the moderate agreement in scores assignment between a professional neuro-radiologist and a primary radiologist, with an ICC ranged 0.65 to 0.75. Additionally, Fabritius showed that manually delineated quantitative perfusion deficit volumes presented good performance for predicting functional outcome in BAO patients (33). However, due to the absence of quantitative thresholds, these studies rely heavily on the image readers’ ability and are time-consuming. Consequently, there is a pressing need for additional knowledge to identify optimal threshold for defining ischemic core that can mimic MRI-DWI in BAO patients.

The RAPID software, frequently used in clinical daily work and numerous clinical studies, including the DEFUSE 3 and DAWN trials, uses rCBF < 30% and Tmax > 6 s as thresholds for defining ischemic core and penumbra, respectively. However, its sensitivity to posterior circulation acute ischemic strokes, particularly brainstem infarction, is limited. Chen explored 116 patients with isolated pontine or midbrain hypoperfusion, of whom 113 were confirmed to have infarction on follow-up MRI, yet none of these lesions could be detected by the RAPID software (34). Studies on ischemic core in the posterior circulation are scarce, with a primary focus on Tmax > 10 s maps generated by RAPID software. Yuen found that the volume of Tmax > 10 s generated by RAPID software was closest to follow-up final infarct volume in BAO strokes after successfully reperfusion (21). Liu further extended these findings, revealing that the perfusion deficit volume of Tmax > 6 s generated by RAPID software presented good predictive performance for outcomes in BAO patients following EVT, outperforming the volume of Tmax > 10 s (35). In our cohort, a limited perfusion deficit volume of Tmax > 10 s, as generated by RAPID software, was significant associated with favorable functional outcomes in the univariate analysis but lost significance in the multivariate analysis, aligning with the findings reported by Sun. (31) A potential explanation for this is the higher rate and timeliness of successful recanalization, which may prevent the penumbra from progressing to infarction. Consistent with prior studies, our findings also underscore that the location of ischemic lesions is more critical than their volume in posterior circulation strokes (17, 36).

In patients with anterior circulation LVO, a conservative threshold (rCBF < 20%) or lower is recommended for estimating the ischemic core, particularly in those who achieve rapid reperfusion following EVT (10). Our findings extend these results and suggest that the pc-ASPECTS based on an ischemic core estimation approach with a CBF threshold of less than 10 mL/100 g/min (rCBF < 20%), generated by Syngo.via software, presented excellent performance in predicting favorable functional outcomes in BAO patients after successful EVT. This method outperformed other promising threshold candidates, such as Tmax >10 s by RAPID software, CBV < 1.2 mL/100 mL by Syngo.via software, and CBF < 15 mL/100 g/min by Syngo.via software, which are more commonly used in studies of intravenous thrombolysis rather than EVT. Consistent with prior researches, in our cohort, the pc-ASPECTS based on Syngo.via ischemic core estimation approaches were lower compared to Tmax >10 s maps by RAPID software (37, 38), indicating the ischemic core extent was more accurately depicted by Syngo.via software determined cores.

Our study aimed to evaluate the ischemic core in patients with BAO by utilizing the absolute values of CBF and CBV. However, this method may result in an underestimation of the ischemic core extent in gray matter and an overestimation in white matter, primarily affecting volume rather than location. Moreover, in cases of LVO strokes, the reductions in CBV typically occur later than those in CBF. Consequently, while color-coded CBV maps provide a more accurate representation of severe ischemia, the sensitivity of CBV-based ischemic core assessments is comparatively lower. This leads to an underestimation of the ischemic core extent when relying solely on CBV measurements. Additionally, further efforts are required to minimize artifacts in core maps generated by Syngo.via software, as these artifacts are the primary cause of discrepancies in score assignments between readers.

Thrombus burden and collateral status have been recognized as predictors of functional outcomes in BAO patients (22–24). Howerer, in our cohort, there was no significance among all CTA scores. One potential explanation is that the importance of these factors may be reduced due to timely and rapidly successful recanalization, which serve as a temporary and reversible pathological state.

## 5 Limitations

Our study has several limitations. First, the retrospective design of this study may introduce bias, and the small sample size from a single comprehensive stroke center necessitates external validation. In our cohort, among 4,543 patients initially suspected of ischemic stroke on admission, 2,831 were ultimately confirmed to have suffered an infarction by follow-up imaging, including 118 cases of basilar artery occlusion (BAO). The incidence rate of BAO was 4.17%, which is higher than the approximately 1% reported in the literature for BAO’s contribution to all ischemic strokes. This discrepancy is primarily due to the fact that many patients with milder symptoms not caused by large-vessel occlusion (such as those with recent subcortical infarctions) either did not undergo CT perfusion examination or sought medical attention later than 24 h after symptom onset. Our stroke center is the largest one in a region with a total population of approximately 2.7 million, and nearly all patients with severe ischemic stroke symptoms seek treatment here. Although the small sample size of BAO in our cohort, this is primarily because many patients, due to prodromal symptoms, were admitted more than 24 h after symptom onset, or their families refused endovascular thrombectomy. After excluding these factors, our data reflects the real-world incidence and treatment scenario of BAO. Second, approximately 35% of basilar artery occlusions are caused by large-artery atherosclerosis. In our cohort, 34.1% of patients underwent intracranial angioplasty and stenting in pursuit of radiological recanalization, which may limit the generalizability of our findings. Additionally, there was a subset of patients with cardiogenic BAO who presented with severe symptoms and had a clear diagnosis but did not undergo CTP examination prior to EVT. Third, our imaging protocol relied on a single CT scanner and software packages, and transferability between vendors and centers requires further validation. Fourth, the thresholds selected to identify the core in our study were based on previous studies of anterior circulation, which may not be the most appropriate for posterior circulation infarction. Additionally, final infarct volume was not measured.

## 6 Conclusion

In conclusion, the pc-ASPECTS based on ischemic core estimation approach with a threshold of CBF < 10 mL/100 g/min (rCBF < 20%) generated by Syngo.via software demonstrated the best predictive value for favorable functional outcomes in BAO patients after successful recanalization, with excellent inter-observer reliability. Future multicenter studies are required to validate our results for clinical decision-making.

## Data Availability

The raw data supporting the conclusions of this article will be made available by the authors, without undue reservation.
